# Suicide and related health risk behaviours among school learners in South Africa: results from the 2002 and 2008 national youth risk behaviour surveys

**DOI:** 10.1186/1471-2458-13-926

**Published:** 2013-10-04

**Authors:** Hilda N Shilubane, Robert AC Ruiter, Bart van den Borne, Ronel Sewpaul, Shamagonam James, Priscilla S Reddy

**Affiliations:** 1 Department of Advanced Nursing Science, University of Venda, Private Bag X 5050, Thohoyandou, South Africa; 2 Maastricht University, Maastricht, The Netherlands; 3 Medical Research Council, Tygerberg, South Africa; 4 Human Sciences Research Council, Pretoria, South Africa

## Abstract

**Background:**

Attempted and completed suicide constitute a major public health problem among young people world-wide, including South Africa (SA). Suicide attempt and completed suicide increase during the adolescent period. One in 5 adolescents considers attempting suicide, but statistics are frequently unreliable.

**Methods:**

Data for this study were derived from the 2002 and 2008 South African Youth Risk Behaviour Surveys (YRBS). The study population comprised grades 8, 9, 10 and 11 students in governmental schools in the nine provinces of SA (N = 10,699 in 2002 and 10,270 in 2008). Key outcome measures were suicide ideation and suicide attempts.

**Results:**

Of the total sample, 18% of the students in 2002 and 19% in 2008 reported to have seriously considered and/or made a plan to commit suicide during the past six months (*Suicide ideation),* whereas 18.5% of students in 2002 and 21.8% in 2008 reported that they had attempted suicide at least 1 time during the past six months. On both suicide measures girls have higher prevalence scores than boys, and older school learners score higher than younger learners. In addition, 32% of the learners reported feelings of sadness or hopelessness. These feelings contributed significantly to the explanation of suicide ideation and suicide attempt next to being the victim or actor in violent acts and illegal substance use.

**Conclusion:**

The prevalence of suicide ideation and suicide attempts among South African adolescents is high and seems to be influenced by a wide spectrum of factors at the demographic, psychological and behavioural level. Hence, more research is needed to determine the behavioural and psychological determinants of suicide among youngsters in order to develop comprehensive intervention strategies for suicide prevention and care.

## Background

Self-destructive behaviours, including suicide, have become a significant public health problem in many parts of the world with South Africa being no exception. Every year almost 1 million people commit suicide resulting in a global suicide rate of 16 per 100,000 people [1]. In the last 45 years suicide rates have increased by 60% worldwide. This increase might be partly caused by better registration procedures in many countries, with data available from 105 countries at the last update in 2011 as compared to 11 countries in 1950 [2]. Whether for example the high rates of suicide in young people reported from India and China in recent years represents a rising trend or an under-reporting bias in older studies is unclear [3]. Suicide is now believed to be among the three leading causes of death among those aged 15-44 years in some countries, and the second leading cause of death in the 10-24 years age group. These figures do not include suicide attempts which are up to 20 times more frequent than completed suicides.

Suicide is complex with psychological, social, biological, cultural and environmental factors involved [1,4]. The emotional, financial and social impact of suicide on the family and friends may last for many years. The prevention of suicide among young people is therefore important. Some studies have shown that legislation which effectively controls access to guns, knives and pesticides ([3,5-8]; Violence Prevention, [1]; WHO, [9]), as well as the prevention and treatment of depression, alcohol and substance abuse, and violence can reduce both homicides and suicides [10,11].

Furthermore, school-based interventions involving self-esteem enhancement and the development of life skills and healthy decision making have been demonstrated to reduce the risk of suicide among the youth [12,13].

### Suicide in South Africa

Whereas suicide prevention programs have been introduced in high income countries, low and middle income countries generally have no prevention programs in place. Moreover, there is a dearth of knowledge about suicide prevalence in the African region. According to the World Health Organization [14], South Africa has suicide prevalence data for 2007. This data shows that adolescents have the highest suicide rate compared to adults and the aged [14]. In South Africa suicide rates are believed to be higher than the worldwide average. According to a national household survey conducted in 2003, the annual suicide rate is 25 deaths per 100,000 people [15]. In a more dated study, Flisher et al., [16] reported suicide deaths to be high in spring or summer and more pronounced among less urbanized and low standard of living. Peltzer and colleagues reported suicide to be the leading causes of death for young people in Limpopo Province in the north-east of South Africa [17]. It is suspected that suicide rates have increased nationwide in more recent years, especially among the youth [18,19]. Furthermore, it is likely that available data underreport the extent of the problem due to insufficient training of police officials in taking detailed history and lack of specialist forensic training among medical practitioners coupled with insufficient resources and opportunities due to social, financial and religious reasons [15,20]. Mental disorders, particularly depression and substance abuse, are associated with more than 90% of all cases of suicide; however, suicide results from many complex socio-cultural factors and is more likely to occur during periods of socioeconomic, family and individual crisis situations, for example the loss of a loved one, unemployment, and humiliation [21,22].

### Youth risk behaviour survey

To the best of our knowledge no recent national representative studies have been conducted on suicide ideation and attempt in adolescents and young people in South Africa. The need for a national survey on health risk behaviours including suicide that is representative of the whole adolescent population is one of the reasons why the South African Medical Research Council (MRC) was commissioned by the national Department of Health of South Africa to undertake the Youth Risk Behaviour Survey in 2002. The terms of reference were to provide representative data on the prevalence of behaviours that place school-going learners at risk that could help in estimating the extent of future potential epidemics, contribute to the development of evidence-based interventions, and provide baseline data against which to compare future interventions. The 2002 YRBS was the first national survey of a cluster of risk behaviours conducted since democracy in South Africa. The second YRBS was conducted in 2008 and aimed to establish the trends over time in the main health risk behaviours [23].

The Youth Risk Behaviour Surveillance System (YRBSS) has been developed by the Centers for Disease Control (CDC) in the United States of America (USA) in 1990 with the aim of monitoring priority health risk behaviours that impact on the main causes of morbidity, mortality and social problems among youth and adults. In the USA, the data have been used to design and evaluate programmes that aim to reduce high risk behaviours. In SA, children and adolescents make up half of the population. There are about 5 670 secondary schools in the country accommodating about 3 514 162 learners at the time of the study in 2002 [24] and 3 831 937 students in 2008 [25]. The school setting therefore provides an appropriate social context to obtain information about young people and their behaviours. This setting is also ideal for the development and implementation of future health interventions.

In the present study we report on suicide ideation and suicide attempt, two precursors of completed suicide, in grade 8 to grade 11 learners in South Africa by comparing national rates with provincial rates for both boys and girls. In addition, relationships are explored between suicide and other health risk behaviours like the use of drugs, alcohol and tobacco, engaging in unsafe sex and violent behaviour. These behaviours typically debut during adolescence and may lead to psycho-social problems like depression and anxiety, which both have been related to suicide (Afifi, Cox, & Katz, [26]).

## Methods

### Research design and sampling procedure

The 2002 and 2008 surveys comprised of cross-sectional national prevalence studies among secondary school learners in South Africa. The study samples comprised of grades 8, 9, 10 and 11 learners (age range: 13-19 years) selected from government schools in the nine provinces of South Africa. The low number of independent or private schools in South Africa, accounting for only 2.2% of the total number of schools, and the difficulty reaching them made it impossible to include these schools in the 2002 survey study. The same barriers and the objective to compare the findings of the two survey rounds excluded private schools also from the 2008 survey. Grade 12 learners were excluded in both surveys to allow learners to focus on their end-of-school examinations. The data collection period ran from August to October in both 2002 and 2008 surveys, and occurred simultaneously in all the nine provinces to avoid seasonal or any other time-related influences.

A two-stage cluster sample design was used to ensure the collection of nationally and provincially representative data from a population arranged into school and class-level clusters. The sole stratum for sampling was the province, which is consistent with the technique adopted by the CDC, where the key level for stratification was the State. At the first stage of sampling, schools were the primary sampling units. For each survey year, a list with all schools in the country was requested from the South African National Department of Education to make sure nationally representative data were used. Schools were selected with a probability proportional to school learner enrolment size in grades 8 to 11. At the second stage of sampling, classes within each participating school were selected systematically with equal probability sampling (with a random start). All learners in the selected classes present at the time of the survey were eligible to participate. It was determined that 1200 learners in each province were to be selected and it was assumed that each class would have approximately 40 learners. Assuming a school and learner participating rate of 80% based on recent experience of youth surveys, 207 schools were initially selected from the nine provinces, amounting to 23 schools per province. During the second stage, on average 2 classes per school were randomly selected. This procedure was the same for both survey rounds except that the sampling in 2008 aimed to address some of the gaps that presented in the 2002 findings, for example, the number of schools with predominantly Indian learners was increased thereby ensuring the national and provincial representativeness of the Indian learner sample. In total 14,766 and 13,379 learners were selected for the 2002 and 2008 surveys respectively, of which 10,699 and 10,270 participated in the surveys. The very large majority of those that did not participate were not present at the time of the survey. Almost none of the learners that were present refused to participate. Additional details about the South African surveys and methods have been reported elsewhere [19,23].

### Study instrument

Data were collected through a self-administered questionnaire drawn from the Youth Risk Behaviour Surveillance System (YRBSS) of the US Centers for Disease Control (CDC) and adapted for the South African context as well as by taking measures of height and weight of each learner.

The 2002 questionnaire was designed to obtain prevalence data from young people on suicide and risk behaviours that affect the current and future health of school learners. The questionnaire was administered using learners’ preferred language/mother tongue. To ensure validity and reliability of the questionnaire pilot tests were conducted among grade 8 learners in five provinces, which enabled the questionnaire to be tested in each of the eleven official languages in South Africa. As a result of expert reviews and the pilot test of the original CDC questionnaire required varying degrees of adaptation, ranging from the addition of new questions, to questions being changed completely, to minor alterations in questions in order to ensure relevance to the South African learners’ risk-taking behaviour and context exposure as well as comparability to surveys conducted locally and internationally. The questions from the original CDC questionnaire referring to behaviour in the past 12 months were changed to refer to only the past 6 months so as not to confound the grade-specific results from the study with behaviour of learners moving through different grades. The 96 items questionnaire included only close-ended questions, without skip patterns or multiple response questions. The questionnaire was further developed in English. Thereafter, groups of three first-language students translated the questionnaire into the remaining 10 official languages in preparation for face and construct validity testing. Based on the testing outcomes, the questionnaire was revised in English and back translated into the remaining 10 official languages by academics proficient in the respective languages. In 2008, the same instrument as used in 2002 was used again besides some small adaptations to better reflect the South African context with regard to specific risk behaviours (e.g., use of illicit drugs) and to reduce long lists of response options.

### Data collection

For each survey year, networks with the schools and the provincial Departments of Health and Education and stakeholders were established to obtain their endorsement and support for the study. A range of individuals from various sectors such as health, education and community structures, for example the Youth Commission, were invited to undergo specialized training in order to be selected as survey administrators in their respective provinces. In 2002 a total of 18 workshops were conducted that trained 510 survey administrators. In 2008, 27 workshops were conducted to train 625 survey administrators. These numbers increased in 2008 due to an Indian over-sample of 21 schools that was performed since the 1^st^ YRBS study conducted in 2002 yielded a very low number of respondents of Indian descent. The responses from these learners were too few to allow meaningful comparisons against learners from other ethnic groups in the main samples of both the 2002 and 2008 surveys^a^.

Following the training, teams with a minimum of 3 members were sent to each school. A team leader was nominated to supervise the survey process of his/her team and to arrange visits to the school. Standardized procedures for conducting the study were developed and described in a script for the survey administrators in English and in the language of the questionnaire. Furthermore, extensive telephonic support was provided to the survey administrators spread across the country during the data collection period. The questionnaires were collected in a class room setting.

Ethical approval for the study was obtained from the South African Medical Association Research and Ethics Committee (SAMAREC). Written permission to conduct the study was obtained from the National Department of Education. Informed consent was obtained from the school principals, parents and learners. Learners were requested not to write their names on the answer sheet to ensure their anonymity. Confidentiality was further maintained by requesting educators to leave the classroom while the trained survey administrators conducted the survey. Further, learners were requested not to communicate with each other or look at the answer sheets of their peers during the completion of the questionnaire. Participation in the study was voluntary. There were no benefits offered to participants. The time taken for a class to complete a questionnaire ranged from 30 minutes to an hour.

### Measures

The same items were used in both surveys to construct the measures below. Scores on items that showed sufficient internal consistency based on Cronbach’s alpha (α) or Pearson correlation (*r*) were averaged into a single index.

### Suicide

Suicide ideation and attempt were assessed by two questions about lifetime suicidal behaviours including contemplation, making a plan and number of attempts. The scores on the binary (1 = *yes*, 0 = *no*) items measuring contemplation (i.e., “During the past 6 months, did you ever seriously consider attempting suicide (that is take some action to end your life)?”) and made a plan (i.e., “During the past 6 months, did you make a plan about how you would attempt suicide (that is take some action to end your life)?”) were averaged to measure *suicide ideation* (2002: *r =* .54, 2008: *r =* .49). The number of *suicide attempts* was measured by one item asking participants “During the past 6 months, how many times did you actually attempt suicide (that is take some action to end your life)?” with answering options 1 = *0 times*, 2 = *1 time*, 3 = *2 or 3 times*, 4 = *4 or 5 times*, 5 = *6 or more times*.

### Psychological measures

One item was included that measured the extent to which learners felt hopeless: “During the past 6 months, have you ever felt so sad or hopeless that you stopped doing some usual activities for two weeks or more in a row?” (1 = *yes*, 0 = *no*).

### Health risk behaviour

Health risk behaviours related to violence, substance abuse, unsafe sex, nutrition, exercise and traffic safety were measured with close-ended questions using 5-point scales unless otherwise indicated.

#### Violence

Items relating to violent behaviour were combined into indices of the extent to which learners felt unsafe, were involved in violent behaviour, carried a weapon, were an actor of violent behaviour, and were a victim of violent behaviour respectively. *Feeling unsafe* during the past month was measured with two items asking learners on how many days they skipped schools because they felt unsafe (a) at school and (b) on their way to school (1 = *never*, 5 = *very often*; 2002: *r* = .58, 2008: *r* = .57). *Carry a weapon* was measured by averaging the scores of two items asking how often learners carried a weapon in-and outside school (1 = *never*, 5 = *very often*; 2002: *r* = .46, 2008: *r* = .47). *Involvement in a physical fight* was measured with two items asking learners how often they were involved in a physical fight *(i.e., hitting, punching*) in-and outside school*;* 1 = *never*, 5 = *very often*; 2002: *r* = .45, 2008: *r* = .45). Being an actor of violent behaviour (*violent behaviour* actor) was measured by four items with answering options 1 = *yes* and 0 = *no* (i.e., During the past 6 months. have you threatened or injured someone with a weapon at school; … have you been a member of a gang; did you ever hit, smack (slap), or physically hurt your boyfriend or girlfriend on purpose; … have you forced someone to have sex when he or she did not want to). Similarly, being a victim of violent behaviour including being bullied (*violent behaviour victim*) was measured by four items coded as 1 = *yes* and 0 = *no* (i.e., During the past 6 months, has someone threatened or injured you with a weapon at school; … have you ever been bullied; … did your boyfriend or girlfriend ever hit, smack (slap), or physically hurt you on purpose; … have you been physically forced to have sex).

#### Substance use

The measures of cigarette use, alcohol use and marijuana use were limited to the past month. One item measured cigarette use (i.e., during the past month, on how many days did you smoke cigarettes; 1 = *never*, 7 = *all 30 days*). Two items measured *alcohol use* by asking learners how often in the past month they had at least one drink of alcohol (e.g., beer, a glass of wine, a ‘tot’ of brandy) and five or more drinks of alcohol on a single occasion (1 = *never*, 5 = *very often*; 2002: *r* = .72, 2008: *r* = .74). One item asked learners how often they had used dagga/hashish (marijuana) in the past month (1 = *never*, 5 = *very often*; *dagga use)*. *Lifetime drug use* was measured by five items that asked how often learners had ever sniffed glue, breathed the contents of aerosol spray cans or inhaled any paint thinners, petrol or benzene and used mandrax, cocaine, heroin and other illegal drugs (e.g., LSD, speed, magic mushrooms) respectively (1 = *never*, 5 = *very often;* 2002: α = .71, 2008: α = .84).

#### Unsafe sex

Having unsafe sex was measured by one item asking learners “When you have sex (when the penis enters the vagina or anus), how often do you or your partner use a condom?” with answering options 1 = *never had sex*; 2 = *never use a condom*; 3 = *rarely use a condom*; 4 = *sometimes use a condom*; 5 = *use a condom most of the time*; 6 = *always use a condom*.

#### Nutrition

One item measured *unhealthy eating* by asking how often respondents ate during the past week fast foods or ‘luxuries’ like a hamburger, fried chicken, “boerewors” roll, hot dog, hot chips, “Gatsby, pies” (1 = *never*, 5 = *very often*).

#### Exercise

Two items measured *physical activity* (0 = did not take part, 7 = 7 days a week) by asking learners how often they exercised or participated in physical activity for at least 20 minutes, such as soccer, netball, rugby and how often they participated in exercise for at least 30 minutes, like fast walking, slow bicycling, and skating (2002: *r* = .36, 2008: *r* = .44).

#### Unsafe driving

Two sets of two items tapped into safety belt use and drunk driving. *Seatbelt* use was measured by averaging the scores on two items that asked learners how often they used a seatbelt when they are in a car or other vehicle driven by (a) themselves and (b) someone else (1 = *never*, 5 = *always*; 2002: *r* = .27; 2008: *r* = .24). Two items asked learners whether they had been in a car or other vehicle (e.g. van, taxi or bus) driven by someone who had been drinking alcohol or whether they themselves had been drinking alcohol (1 = *never*; 5 = *very often (six or more time)*; 2002: *r* = .31, 2008: *r* = .22).

#### Suicide hospitalization

Was measured by one item using the question “ if you attempted suicide during the past 6 months, did any attempt result in an injury, poisoning, or overdose that had to be treated by a doctor or a nurse” and the response options were ( 1 = *I did not attempt*; 2 = *Yes* and 3 *= No*).

### Demographic measures and school performance

At the learner level, measures were taken of gender (0 = *boy*; 1 = *girl*), age, race, school level, school performance, and family income. *Age* was calculated from the birth date that learners provided. *Race* was measured by asking learners to classify themselves as either (1) *Black*, (2) *Coloured*, (3) *Indian*, (4) *White* or (5) *Other*.^b^*School level* was coded as (1) grade 8, (2) grade 9, (3) grade 10 and (4) grade 11. Monthly family income was measured by two items that asked for the employment status of the father and mother of the learners, respectively (i.e., “Does your father [mother] have a paid job? (paid job also refers to those who are self-employed e.g. your father has a shop at home)”. The answers were recoded to 1 = *no/I don’t know/my father/mother is dead*, 2 = *yes, works less than 5 days a week*, 3 = *yes, works 5 or more days a week* after which the scores on the two items were averaged to form an index of *family income*. *School performance* was measured with the item “During the past 6 months, how would you describe your grades in school?” with answering options: 1 = *mostly F’s (< 40%)*, 2 = *mostly E’s (40-49%)*, 3 = *mostly D’s (50-59%)*, 4 = *mostly C’s (60-69%)*, 5 = *mostly B’s (70-79%)*, 6 = *mostly A’s (80% or more)*.

### Data analysis

Statistical analyses were done using the software program SPSS version 21. Because of strong positively skewed distributions (i.e., majority of the scores in the lower range of the scales) measures related to violent behaviour and substance use and the individual measures of unsafe sex and drunk driving were dichotomized into 0 = *never/inconsistent* and 1 = *ever/consistent*. For the same reason, the measure of suicide attempt was dichotomized (0 = *no attempt*, 1 = *1 or more attempts*).

Various methods were used to describe and analyze the data, among which cross tabulations with frequencies and chi-square tests for comparing boys and girls on the outcome measures and calculating the national and provincial prevalence of suicidal behaviours (suicide ideation, making a plan, suicide attempts); independent samples *t* tests and chi-square tests to compare suicidal behaviours between males and females and among provinces; and stepwise linear and logistic regression analyses to determine the association between suicide ideation and suicide attempt respectively and demographic measures (age, gender, school performance, monthly family income) in the first step, psychological measures in the second step, and health risk behaviours in the third step. For each survey year, these regression analyses were conducted for the total sample as well as for boys and girls separately. In relation to the large sample size, the significance level was set at *p* < .01 for all analyses.

## Results

Table 1 provides the descriptive scores on the demographic and outcomes measures (suicide, psychological measures, and health risk behaviours) for the total sample and for boys and girls separately for the 2002 and 2008 surveys.

**Table 1:** Descriptive scores on demographic and outcome measures for the 2002 and 2008 samples and comparing boys versus girls

	**Total**		**Boys**		**Girls**		**Test statistic (boys vs. girls)**	
	**2002**	**2008**	**2002**	**2008**	**2002**	**2008**	**2002**	**2008**
	**(N = 10549)**	**(N = 10097)**	**(N = 4929)**	**(N = 4949)**	**(N = 5620)**	**(N = 5148)**		
Outcome measure								
**Demographic variables**								
*Age (M, SD)*	15.98 (1.80)	16.08 (1.69)	16.20 (1.79)	16.25 (1.69)	15.79 (1.79)	15.89 (1.66)	*t*(10017) = 11.67^**^	*t*(9836) = 10.89^**^
*Race (*%*)*							χ^2^(df = 4, N = 10450) = 5.08	χ^2^(df = 4, N = 10050) = 15.42
Black	74.1	78.8	74.7	78.6	73.5	79.0		
Coloured	15.0	14.3	14.4	13.7	15.6	14.9		
Indian	1.3	0.8	1.4	0.9	1.2	0.8		
White	8.7	5.2	8.4	6.1	8.8	4.4		
Other	0.9	0.8	1.0	0.8	0.9	0.9		
*Province (*%*)*							χ^2^(df = 8, N = 10549) = 25.59^**^	χ^2^(df = 8, N = 10097) = 21.24^*^
North West	11.3	9.4	12.5	9.4	10.2	9.5		
Gauteng	11.0	12.4	10.9	12.8	11.1	11.9		
Free State	10.7	9.4	11.1	9.2	10.4	9.6		
Limpopo	9.5	9.5	9.7	9.4	9.4	9.7		
Mpumalanga	11.9	11.5	12.2	11.6	11.7	11.3		
Kwazulu-Natal	11.2	12.3	11.0	12.3	11.5	12.3		
Eastern Cape	10.8	11.2	9.9	11.8	11.6	10.7		
Northern Cape	10.0	12.5	9.8	13.0	10.3	12.1		
Western Cape	13.5	11.8	13.0	10.5	14.0	13.0		
*School level (*%*)*							χ^2^(df = 3, N = 10549) = 24.87^**^	χ^2^(df = 3, N = 10097) = 5.35
Grade 8	27.2	23.5	28.0	24.1	26.5	23.0		
Grade 9	34.0	24.0	34.5	23.2	33.5	24.7		
Grade 10	22.5	29.0	23.1	29.6	22.1	28.4		
Grade 11	16.3	23.5	14.4	23.1	17.9	23.9		
*School performance (M, SD)*	4.10 (1.36)	4.00 (1.40)	4.11 (1.35)	3.93 (1.40)	4.09 (1.36)	4.04 (1.40)	*t*(8875) = 0.37	*t*(8875) = −3.44^**^
*Employment parents (*%*)*							χ^2^(df = 2 N = 10392) = 1.23	χ^2^(df = 2 N = 9901) = 20.93^**^
-Father								
Unemployed	50.8	52.4	50.3	50.4	51.4	54.5		
Parttime	8.5	8.3	8.6	9.2	8.5	7.4		
Fulltime	40.6	39.3	41.1	40.5	40.2	38.1		
-Mother							χ^2^(df = 2 N = 10420) = 10.32^*^	χ^2^(df = 2 N = 9962) = 4.65
Unemployed	11.8	12.4	12.6	12.7	11.1	12.1		
Parttime	31.2	31.6	29.8	32.4	32.4	30.8		
Fulltime	57.0	56.0	57.6	54.9	56.6	57.1		
**Suicide**								
*Suicide ideation (M, SD)* Nationwide	.18 (.34)	.19 (.34)	.16 (.32)	.17 (.33)	.20 (.36)	.21 (.35)	*t*(10454) = 5.99^**^	*t*(10016) = 5.14^**^
North West	.15 (.32)	.17 (.33)	.13 (.29)	.16 (.33)	.17 (.34)	.18 (.33)	*t*(1175) = 2.54^*^	*t*(943) = 0.67
Gauteng	.20 (.35)	.19 (.33)	.16 (.31)	.17 (.31)	.23 (.37)	.20 (.35)	*t*(1149) = 3.32^**^	*t*(1245) = 1.64
Free State	.20 (.35)	.18 (.34)	.18 (.33)	.13 (.30)	.22 (.37)	.22 (.37)	*t*(1123) = 2.01	*t*(934) = 3.92^**^
Limpopo	.18 (.33)	.19 (.34)	.18 (.33)	.17 (.32)	.19 (.33)	.21 (.36)	*t*(995) = 0.08	*t*(953) = 1.92
Mpumalanga	.21 (.34)	.21 (.35)	.18 (.33)	.21 (.35)	.23 (.36)	.22 (.35)	*t*(1245) = 2.61^*^	*t*(1153) = 0.50
Kwazulu-Natal	.18 (.34)	.18 (.33)	.17 (.34)	.18 (.33)	.19 (.35)	.18 (.33)	*t*(1166) = 0.65	*t*(1230) = 0.12
Eastern Cape	.15 (.32)	.19 (.34)	.17 (.33)	.17 (.32)	.14 (.31)	.21 (.36)	*t*(1126) = 1.58	*t*(1112) = 1.85
Northern Cape	.19 (.35)	.21 (.37)	.15 (.32)	.20 (.34)	.22 (.37)	.20 (.34)	*t*(1048) = 2.98^*^	*t*(1253) = 0.26
Western Cape	.18 (.34)	.21 (.37)	.13 (.30)	.16 (.32)	.22 (.37)	.25 (.39)	*t*(1411) = 4.70^**^	*t*(1177) = 4.50^**^
*Suicide attempt (*%*)*								
Nationwide	18.5	21.8	17.3	20.8	19.5	22.7	χ^2^(df = 1, N = 10498) = 8.37^*^	χ^2^(df = 1, N = 10028) = 5.54
North West	18.3	21.0	17.5	21.5	19.2	20.6	χ^2^(df = 1, N = 1182) = 0.57	χ^2^(df = 1, N = 947) = 0.11
Gauteng	16.5	21.5	13.8	20.9	18.8	22.1	χ^2^(df = 1, N = 1159) = 5.30*	χ^2^(df = 1, N = 1244) = 0.28
Free State	20.8	18.5	20.5	15.8	21.2	21.1	χ^2^(df = 1, N = 1128) = 0.08	χ^2^(df = 1, N = 944) = 4.34
Limpopo	20.3	21.7	19.7	20.7	20.9	22.6	χ^2^(df = 1, N = 999) = 0.24	χ^2^(df = 1, N = 953) = 0.50
Mpumalanga	22.9	24.4	20.4	22.5	25.1	26.3	χ^2^(df = 1, N = 1251) = 3.93	χ^2^(df = 1, N = 1156) = 2.28
Kwazulu-Natal	16.5	22.5	14.9	23.7	17.9	21.3	χ^2^(df = 1, N = 1180) = 1.89	χ^2^(df = 1, N = 1231) = 1.00
Eastern Cape	15.0	22.9	16.0	22.5	14.2	23.4	χ^2^(df = 1, N = 1123) = 0.70	χ^2^(df = 1, N = 1112) = 0.15
Northern Cape	18.2	21.5	16.8	20.0	19.3	23.0	χ^2^(df = 1, N = 1057) = 1.10	χ^2^(df = 1, N = 1256) = 1.66
Western Cape	17.8	21.4	16.0	18.4	19.2	23.6	χ^2^(df = 1, N = 1419) = 2.41	χ^2^(df = 1, N = 1185) = 4.70
*Suicide hospitalization (%)*								
Nationwide	19.9	20.0	21.3	20.6	18.6	19.4	χ^2^(df = 1, N = 5511) = 6.38*	χ^2^(df = 1, N = 5069) = 1.12
North West	18.4	23.9	17.2	23.3	19.8	24.6	χ^2^(df = 1, N = 625) = 0.72	χ^2^(df = 1, N = 435) = 1.00
Gauteng	17.1	19.5	18.7	21.4	15.9	17.6	χ^2^(df = 1, N = 630) = 0.91	χ^2^(df = 1, N = 564) = 1.09
Free State	18.3	15.6	18.2	11.6	18.4	19.1	χ^2^(df = 1, N = 585) = 0.03	χ^2^(df = 1, N = 495) = 5.28
Limpopo	27.7	22.4	28.1	20.0	27.3	24.9	χ^2^(df = 1, N = 520) = 0.04	χ^2^(df = 1, N = 460) = 1.58
Mpumalanga	24.9	24.7	24.8	23.2	25.1	26.4	χ^2^(df = 1, N = 694) = 0.01	χ^2^(df = 1, N = 611) = 0.83
Kwazulu-Natal	21.1	21.1	21.5	26.3	20.8	15.4	χ^2^(df = 1, N = 516) = 0.04	χ^2^(df = 1, N = 626) = 11.18^**^
Eastern Cape	21.4	17.8	25.0	20.2	18.3	15.1	χ^2^(df = 1, N = 495) = 3.33	χ^2^(df = 1, N = 641) = 2.78
Northern Cape	17.2	18.8	19.0	19.7	15.7	17.8	χ^2^(df = 1, N = 598) = 1.09	χ^2^(df = 1, N = 605) = 0.33
Western Cape	15.7	20.0	21.2	18.1	11.0	17.2	χ^2^(df = 1, N = 848) = 16.61^**^	χ^2^(df = 1, N = 632) = 0.08
**Psychological measures**								
Feeling hopeless (%)	32.5	32.5	31.6	32.6	33.4	32.4	χ^2^(df = 1, N = 8009) = 3.07	χ^2^(df = 1, N = 7447) = 0.03
Body dissatisfaction (M, SD)	2.80 (0.90)	2.71 (0.95)	2.65 (0.87)	2.57 (0.94)	2.93 (0.91)	2.85 (0.93)	*t*(8310) = 14.02^**^	*t*(7650) = 13.16^**^
Feeling unsafe (%)	36.1	34.5	36.9	32.9	35.4	33.7	χ^2^(df = 1, N = 10521) = 2.77	χ^2^(df = 1, N = 10073) = 2.92
**Health risk behaviours**								
*Violence (%)*								
Carry a weapon	21.3	17.8	33.1	26.5	11.0	9.5	χ^2^(df = 1, N = 10513) = 761.81^**^	χ^2^(df = 1, N = 10080) = 499.34^**^
Involvement physical fight	39.0	37.3	47.3	45.6	31.8	29.4	χ^2^(df = 1, N = 10522) = 263.69^**^	χ^2^(df = 1, N = 10073) = 282.76^**^
Violent behaviour victim	55.6	53.6	56.1	55.4	55.2	51.9	χ^2^(df = 1, N = 10538) = 0.81	χ^2^(df = 1, N = 10086) = 12.54^***^
Violent behaviour actor	30.6	35.1	37.0	41.7	25.0	28,8	χ^2^(df = 1, N = 10521) = 177.46^**^	χ^2^(df = 1, N = 10082) = 183.62^**^
*Substance use (%)*								
Smoking	25.0	21.9	31.8	27.7	19.2	16.4	χ^2^(df = 1, N = 10398) = 219.02^**^	χ^2^(df = 1, N = 10009) = 188.04^**^
Alcohol	37.1	41.0	43.5	47.5	31.4	34.7	χ^2^(df = 1, N = 10541) = 163.07^**^	χ^2^(df = 1, N = 10047) = 170.62^**^
Marijuana	10.0	9.8	14.9	13.7	5.8	6.0	χ^2^(df = 1, N = 10471) = 242.30^**^	χ^2^(df = 1, N = 9966) = 166.72^**^
Illegal drugs	22.4	20.2	26.4	25.2	18.9	15.2	χ^2^(df = 1, N = 10460) = 85.13^**^	χ^2^(df = 1, N = 9540) = 146.83^**^
*Unsafe sex (%)*	64.0	55.1	64.3	57.0	63.5	52.4	χ^2^(df = 1, N = 4217) = .29	χ^2^(df = 1, N = 3813) = 7.962^**^
*Unhealthy eating (M, SD)*	3.22 (1.24)	3.22 (1.26)	3.20 (1.28)	3.23 (1.29)	3.24 (1.21)	3.21 (1.24)	*t*(10291) = 1.56	*t*(9618) = 0.59
*Exercise (M, SD)*	2.95 (2.20)	2.78 (2.21)	3.30 (2.22)	3.07 (2.25)	2.64 (2.13)	2.50 (2.12)	*t*(10477) = 15.47^**^	*t*(9918) = 12.85^**^
*Unsafe driving*								
Seatbelt use (M, SD)	2.44 (1.34)	2.75 (1.47)	2.46 (1.31)	2.84 (1.42)	2.42 (1.37)	2.66 (1.51)	*t*(10513) = 1.46	*t*(10513) = 1.46
Drunk driving (%)	38.0	41.9	43.2	46.8	33.4	37.3	χ^2^(df = 1, N = 10495) = 107.04^**^	χ^2^(df = 1, N = 10084) = 93.62^**^

^*^p < .01; ^**^p < .001.

### Demographic characteristics of the sample

In both boys and girls, those that identified themselves as Black formed a large majority of the total samples in 2002 and 2008 reflecting the racial distribution of the SA population. The 11 provinces of South Africa were rather equally represented with some overrepresentation of Western Cape Province in 2002, and under-representation of the lower populated provinces Limpopo, North West and Free State in 2008. In the 2002 survey, students in grade 11 were somewhat under-represented compared with the number of students in the other grades.

### Suicide

#### Suicide ideation

Nationwide, 18% of the students in 2002 and 19% in 2008 indicated to have seriously considered and/or made a plan to commit suicide during the past six months. This number was significantly higher for girls than for boys in both surveys (see Table 1). At the provincial level, the gender difference was significant in five out of the nine provinces in 2002 (i.e., North West, Gauteng, Mpumalanga, Northern Cape, Western Cape; *p*s < .01), whereas this pattern was maintained for only two provinces in 2008 (i.e., Free State, Western Cape).

#### Suicide attempt

The data pattern of suicide attempts showed that at the national level the percentage of students that have attempted suicide at least 1 time during the past six months was higher in 2008 (21.8%) than in 2002 (18.5%). At the national level, this number was significantly higher for girls than for boys in both 2002 and 2008 surveys. At the provincial level this gender difference was only significant in Gauteng province in 2002. Across gender groups, suicide attempt seems to be somewhat more prevalent in Mpumalanga compared with the other provinces.

#### Suicide hospitalization

Among those that reported to have attempted suicide, about 20% were hospitalized. This proportion was the same across survey rounds with boys somewhat more likely to be hospitalized than girls, but only in 2002 this difference was significance.

### Psychological measures

Nationwide, 32.5% of the students in both 2002 and 2008 indicated to have felt sad or hopeless in the last six months to such an extent that they stopped some usual activities for two weeks or more in a row. No significant difference on feelings of hopeless was found between boys and girls in both surveys. Concerning body weight, students reported on average that they had about the right weight, feeling more underweight than overweight and thus seemed to be satisfied with their body. Boys reported to feel somewhat more underweight than girls in both 2002 and 2008. Nationwide, 36.1% of the students in 2002 and 34.5% in 2008 felt unsafe at school or on their way to school in the past month. No differences on this measure were found between boys and girls.

### Health risk behaviours

#### Violence

Measures of violent behaviours show that the prevalence of violence is high. Across both surveys, about a fifth of the students indicated to have carried a weapon in-or outside school in the month preceding the survey. Furthermore, more than a third of the students reported to have been involved in a physical fight, whereas more than 50% reported to have been a victim of violent behaviour (including bullying) in the past six months. About 40% of the boys and 25% of the girls were involved as actor in violent behaviour. On all but one of the violence measures these numbers were significantly higher for boys than for girls.

#### Substance use

The national prevalence rates for past month smoking, alcohol use and marijuana use, and lifetime use of illegal drugs was respectively 25, 37, 10 and 22 percent in 2002 and 22, 41, 10, and 20 percent in 2008. On all measures boys scored significantly higher than girls in both 2002 and 2008.

#### Unsafe sex

Of those who ever had sex, 64% reported to have had at least one time unsafe sex in their lifetime in 2002 whereas this percentage dropped significantly to 55% in 2008, with more boys reporting to have been unsafe than girls but only in 2008.

#### Unhealthy eating and exercising

No differences were found with regard to unhealthy eating behaviour across surveys and gender groups. Girls reported however less exercise behaviour than boys in both 2002 and 2008, and reported exercise was lower in 2008 than in 2002.

#### Unsafe driving

Seatbelt use increased between 2002 and 2008, but so did drunk driving. In 2002 and 2008, boys reported significantly more seatbelt use than girls. Boys also reported more drunk driving in the past month.

### Determinants of suicide ideation

Stepwise linear regression analysis was conducted to determine the unique contribution of the demographic, psychological and health risk behaviour measures to the amount of variance explained in suicide ideation (see Table 2). Demographic measures were included in the model in step 1, the two psychological measures in step 2, and the risk behaviour measures at step 3. At each step, variables were included simultaneously with the ENTER method.

**Table 2:** **Hierarchical linear regression of suicide ideation on demographic, psychological and behavioural measures for the 2002 and 2008 samples presenting standardized regression coeffients (beta), significance values, and explained variance (R**^**2**^**)**

	**Block 1**		**Block 2**		**Block 3**	
	**2002**	**2008**	**2002**	**2008**	**2002**	**2008**
Block/Variables entered	Beta	Beta	Beta	Beta	Beta	Beta
**Demographic measures**						
Age	.096^**^	.129^**^	.04^*^	.068^**^	.016	.039
Gender	.091^**^	.086^**^	.08^**^	.088^**^	.129^**^	.125^**^
Grade	-.038^*^	-.114^**^	-.020	-.082^**^	-.005	-.054^*^
School performance	-.004	-.026	.015	-.020	.02	-.012
Income father	-.005	-.005	.004	.006	-.005	.005
Income mother	-.010	.047^*^	-.003	.037	-.014	.022
**Psychological measures**						
Feeling hopeless			.269^**^	.247^**^	.21^**^	.197^**^
Body dissatisfaction			-.006	-.047^**^	.008	-.025
Feeling unsafe			.12^**^	.11^**^	.067^**^	.050^**^
**Risk behaviour measures**						
*Violence*						
Carry a weapon					.024	.013
Involvement in physical fight					.032	.017
Violent behaviour-victim					.072^**^	.091^**^
Violent behaviour-actor					.081^**^	.086^**^
*Substance use*						
Smoking					.036	.073^**^
Alcohol					.053^**^	.015
Marijuana					.046^*^	.038
Illegal drugs					.054^**^	.057^**^
*Unsafe sex*					.019	-.002
*Unhealthy eating*					.020	.030
*Exercise*					.009	-.009
*Unsafe driving*						
Seatbelt use					-.005	.016
Drunk driving					.008	.019
**R**^**2**^	.013	.016	.103	.098	.150	.146
**R**^**2 **^**change**	.013	.016	.091	.082	.047	.048
**F change**	11.019**	12.512^**^	171.412^**^	136.628^**^	21.464^**^	19.647^**^

*p<.01; **<.001.

For 2002, including the demographic variables at step 1 only explained 1% of the variance in suicide ideation. Adding the psychological measures at step 2 increased the explained variance to 10%. Including the risk behaviour measures further increased the explained variance to 15%. The final regression model was significant and indicated significant positive contributions for gender (female > male), feelings of hopelessness, feeling unsafe, being an actor or victim of violent behaviour, and substance use including alcohol, marijuana and illegal drugs. The remaining measures showed no significant contributions to the prediction of suicide ideation.

For 2008, a similar pattern of findings emerged with psychological measures increasing the explained variance to 10% and risk behaviour measures adding another 5%, resulting in a total explained variance of 15%. In the final model, the same measures showed unique positive contributions to the prediction of suicide ideation except that smoking behaviour now showed a significant contribution whereas alcohol and marijuana did not. Furthermore, grade level was negatively associated with suicide ideation in the final model.

### Determinants of suicide attempt

Stepwise logistic regression analyses were conducted to identify significant associations between the demographic, psychological and risk behaviour measures and suicide attempt by building again a hierarchical model (see Table 3).

**Table 3:** **Hierarchical logistic regression of suicide attempt on demographic, psychological and behavioral measures for the 2002 and 2008 samples presenting odds ratio’s (OR), significance values and explained variance (R**^**2**^**)**

	**Block 1**		**Block 2**		**Block 3**	
	**2002**	**2008**	**2002**	**2008**	**2002**	**2008**
**Block/Variables entered**	**Odds ratio**	**Odds ratio**	**Odds ratio**	**Odds ratio**	**Odds ratio**	**Odds ratio**
**Demographic measures**						
Age	1.176^**^	1.277**	1.095^**^	1.174^**^	1.034	1.126^**^
Gender	1.411^**^	1.354**	1.413^**^	1.418^**^	2.178^**^	2.095^**^
Grade	0.736^**^	0.690**	0.781^**^	0.748^**^	.819^**^	0.790
School performance	0.968	0.938	0.995	0.943	0.999	0.959
Income Father	0.910	1.026	1.006	1.059	0.968	1.050
Income Mother	0.035	1.007	0.933	0.994	0.894	0.946
**Psychological measures**						
Feeling hopeless			2.459^**^	2.243^**^	1.752^**^	1.755^**^
Body dissatisfaction			.980	0.829^**^	1.029	0.856^**^
Feeling unsafe			2.459^**^	2.096^**^	1.655^**^	1.419^**^
**Risk behaviour measures**						
*Violence*						
Carry a weapon					1.284	1.474^**^
Involvement in physical fight					1.358^**^	1.323^**^
Violent behaviour-victim					1.563^**^	1.476^**^
Violent behaviour-actor					1.808^**^	1.431^**^
*Substance use*						
Smoking					1.176	1.579^**^
Alcohol					1.332^**^	1.159
Marijuana					1.500^**^	1.095
Illegal drugs					1.390^**^	1.576^**^
*Unsafe sex*					1.310^**^	1.160
*Unhealthy eating*					1.064	1.020
*Exercise*					1.025	1.023
*Unsafe driving*						
Seatbelt use					1.003	1.009
Drunk driving					1.231	1.210
**R**^**2**^	.02		.11		.21	
**R**^**2 **^**change**	.02		.09		.10	
**χ**^**2 **^**change**	68.49**		276.78^**^		341.27^**^	

*p<.01; **<.001.

For 2002, the final model provided significant positive contributions for gender (female > male), feeling hopelessness, feeling unsafe, being involved in physical fights, being a victim or actor of violent behaviour, using alcohol, marijuana and illegal drugs, and having unsafe sex. A negative association was found for grade level.

In 2008, the same relationships were found except that grade level, alcohol and marijuana use and having unsafe sex were no longer significant, while age, carrying a weapon and smoking now showed a significant positive association and body dissatisfaction a negative association. The explained variance in suicide attempt was about 21% for both 2002 and 2008^c^.

## Discussion

The Youth Risk Behaviour Survey is the largest public health surveillance system in South Africa monitoring a broad range of health-risk behaviours among high school students. The results of two national representative surveys on health risk behaviours of South African adolescents indicate that 18% of the students in 2002 and 19% in 2008 seriously considered and made a plan to commit suicide. This number was significantly higher for girls than for boys across both surveys. Suicidal attempt followed the pattern of suicide ideation, 18.5% of learners attempted suicide at least once. Similarly, girls had a significantly higher percentage (19.5%) than boys (17.3%).

Previous studies conducted in South Africa [20,27,28] and elsewhere [1,29] showed a higher suicide rate in male adolescents than female. In contrast, the present study found a higher rate of suicide ideation and attempt among females than male school learners. Our findings are consistent with some previous studies conducted in South Africa [30-32] and elsewhere [33-36] that show that suicide ideation and attempts are higher among female adolescents than male. This difference between more suicidal deaths in boys and more ideation and attempt in girls may be explained by evidence that suggests that girls rather use non-lethal means to end their pain or signal others that they are unhappy [37]. This might also explain why in our findings boys reported being more frequently admitted to hospital than girls following a suicide attempt. Also fear of stigma might cause young people not to consult health professionals following a suicide attempt.

In the present study, the suicide attempts rate seems to be slightly higher than suicide ideation. This is supported by previous studies that found an increasing prevalence of impulsive acts of self-harm; that is, acts that are not accompanied by a specific plan or even thoughts of killing oneself. These unplanned attempts constitute an important subtype of suicidal behaviour among adolescents and comprise a substantial proportion of medically serious attempts [38,39].

Nationwide, a high rate of learners felt hopeless with no significant difference between the two genders. Feelings of hopelessness were found to contribute significantly to the explanation of both suicide ideation and attempt in both survey rounds. This is not surprising given the positive relationship between depression and suicidal behaviours that have been repeatedly reported in studies conducted in South Africa as well as in other countries [40,19,44]. Analyses further revealed that frequent health risk behaviours such as violence and substance use were significantly associated with suicide ideation and attempt. Boys scored higher on these measures than girls. It is possible that substance use may serve to increase hopelessness and thus suicidal ideation and attempt among learners. Different studies support this finding and also demonstrate positive and significant relations between use of drugs and suicidal behaviour ([45,46]; Epstein et al., [47,48]).

In the present study we did not find a univariate association between forced sex and suicidal behaviour, although Sorsdahl, Stein, Williams and Nock [49] in their study of traumatic events and suicidal behaviour found sexual violence as a predictor of lifetime suicide attempts. Also, we found unique explanations of suicidal behaviour by feelings of unsafety at school (2002 and 2008) and body image (only 2008). These findings suggest that the promotion of safe school environments context and personal variables such as self-esteem might be good intervention targets for future prevention programmes, next to well documented predictors such as feelings of sadness and hopelessness.

The study increased our knowledge of adolescent suicidal behaviour, but has some limitations. Data collection used a self-completion questionnaire. It is possible that some study participants may have misreported on their risk behaviours. Only students attending school participated in the survey, leaving out the drop-outs. Thus caution should be taken in generalizing the findings to the general population of adolescents. Furthermore, due to the cross-sectional nature of our study no conclusions about causality can be made. The usual interval for YRBS in the US is 2 years. In our study the interval between the two surveys was six years. This long interval made it difficult to study within-participant changes and trends over time in a same cohort of learners. Furthermore, both cohorts did not include grade 12 learners, whereas evidence for the US and South Africa suggest that suicidal rates appear to be higher in years of school transition [23,50]. Future YRBS studies in South Africa might aim to reduce the inter-study time interval and include all grades, for example by moving data collection to the first semester of the school year. Finally, we did not explore the role of ethnicity in explaining suicide in our adolescent population. Evidence from the US suggests that suicide rates might differ as a function of ethnicity [51,52].

The study demonstrated that there is an increase in suicide attempts in the absence of suicide ideation, and that a high rate of learners felt hopeless. The result from our study suggests that evidence-and theory-based suicide prevention programs should be developed to reduce the high suicide rate of (mainly black) school learners in South Africa. The school curriculum content should include information on mental health and suicide. The prevalence of depression and thus suicide ideation in young people should be reduced by early identification of at risk students, for example, those experiencing forced sexual encounters, poor social support, and previous suicide attempts [44]. The Government and non-governmental organizations should work collaboratively in providing in-service education to health promoters and school teachers to address risk behaviours in an integrated and comprehensive manner, for example within the WHO concept of health promoting schools [53]. The department of Health and the department of Education should work together in reviewing the content included in the Life Orientation (LO) subject. This will ensure that service providers and schools do not feel overwhelmed and overburdened by multiple single behaviour interventions but approach suicidal behaviour and related risk behaviours in a comprehensive manner, guided by systematic explorative and confirmative research to develop evidence- and theory-based suicide prevention and behaviour change programmes using intervention development frameworks such as Intervention Mapping [54].

## Conclusion

The prevalence of suicide ideation and suicide attempts among South African adolescents is high and seems to be influenced by a wide spectrum of factors at the demographic, psychological and behavioural level. More research is needed to determine the behavioral and psychological determinants of suicide among youngsters in order to develop comprehensive intervention strategies for suicide prevention and care.

## Endnotes

^a^The additional sample of mainly Indian learners in 2008 was not included in the analyses reported here. For a complete report of the 2008 survey findings including the Indian over-sample, see Reddy et al., [23].

^b^During the Apartheid years all South Africans were classified in accordance with the Population Registration Act of 1950 into “racial groups” viz. “Black/African” (people mainly of African decent), “Coloured” (people of mixed decent), “White” (people mainly of European decent), or “Indian” (people mainly of Indian decent). The provision of services occurred along these “racially” segregated lines. The disproportionate provision of services to different “race groups” led to inequities. Information is still collected along these “racial” divisions in order to redress these inequities. In no way do the authors subscribe to this classification.

^c^The four regression analyses were repeated with hierarchical linear modeling analyses to control for the supposedly random influence of school membership. These analyses yielded the exact same patterns of findings for both survey rounds and on both outcomes measures. We stayed with the stepwise regression approach to demonstrate the contributions of the three categories of predictors in the explanation of suicide ideation and suicide attempt in both surveys.

## Competing interests

The authors declare that they have no competing interests.

## Authors’ contributions

HNS (*University of Venda, South Africa)–*was involved in research instrument development, data collection, data analysis and wrote the paper. SJ (Medical Research Council, South Africa*)–*was involved in research instrument development, data collection and writing of the paper. RS (Medical Research Council, South Africa*)–*was involved in data preparation and analysis. RACR (*Maastricht University, the Netherlands*)–was involved in conceptualization of the paper, data analysis and writing of the paper. BVDB (*Maastricht University, the Netherlands)*–was involved in conceptualization of the paper, data analysis and writing of the paper. PSR (*Human Sciences Research Council Cape Town, South Africa)–*project leader of SA YRBS studies and involved in research instrument development. All authors read and approved the final manuscript.

## Pre-publication history

The pre-publication history for this paper can be accessed here:

http://www.biomedcentral.com/1471-2458/13/926/prepub
